# Body fat percentiles of Brazilian adolescents according to age and sexual maturation: a cross-sectional study

**DOI:** 10.1186/1471-2431-13-96

**Published:** 2013-06-19

**Authors:** Isa de Pádua Cintra, Gerson Luis de Moraes Ferrari, Ana Carolina de Sousa Vieira Soares, Maria Aparecida Zanetti Passos, Mauro Fisberg, Maria Sylvia de Souza Vitalle

**Affiliations:** 1 Adolescence Division, Pediatric Department, Universidade Federal de São Paulo (Federal University of São Paulo), Rua Botucatu, 715 Vila Clementino, São Paulo, Brazil; 2 Centro de Estudos do Laboratório de Aptidão Física de São Caetano do Sul (Physical Fitness Research Center of São Caetano do Sul), São Caetano do Sul, Brazil

**Keywords:** Body fat percentiles, Adolescents, Adiposity, Sexual maturation, Skinfold thickness

## Abstract

**Background:**

The objective of this study was to determine body fat percentiles of adolescents in the city of São Paulo, Brazil, according to gender, age, and sexual maturation.

**Methods:**

This study involved 4,690 adolescents aged 10–15 years across 31 schools in the city of São Paulo. Sexual maturation was assessed in terms of Tanner stage. The body fat percentage was calculated using skinfold thickness. Percentile curves were calculated using the LMS (curve, mean, and coefficient of variation) method.

**Results:**

The mean body fat percentages were lower in boys aged 10–12 and 13–15 years than in girls. Body fat percentages decreased progressively with sexual maturation in boys, but increased in girls. The 85th, 95th, and 97th percentiles represent the cutoff points for moderately elevated, elevated, and very elevated body fat percentages, respectively, in pre-pubescent boys (85th, 95th, and 97th percentiles: 32.54, 95 41.04, and 97, respectively) and pubescent boys (31.09, 36.30, and 44.33, respectively). These cutoff points were lower in pre-pubescent girls (29.52, 35.01, and 41.82, respectively) and in the 97th percentile in pubescent girls (31.55, 36.20, and 41.86, respectively).

**Conclusion:**

To our knowledge, these are the first body fat percentages cutoff points according to sexual maturation for adolescents aged 10–15 years in Brazil. Our results provide a significant contribution to the assessment of body composition in this population.

## Background

Obesity has become a serious public health problem that affects people of all ages, gender, races, and socioeconomic levels. Its prevalence has increased worldwide in recent decades, including in developing countries like Brazil [1,2].

Excess body fat is associated with risk factors such as diabetes, hypertension, and elevated triglyceride and cholesterol levels in children and adolescents. Consequently, obesity is associated with the early onset of cardiovascular diseases, and increased risk of morbidity and mortality in adulthood [3-6].

During the growth stage, there is some variability in body fat percentages, meaning subcutaneous and abdominal fat distribution change with age [7].

Body fat composition and distribution differ between gender. During puberty, for example, hormones induce pronounced sexual dimorphism, as boys show greater muscle mass gain than fat mass gain, whereas girls show greater fat mass gain as a natural part of their sexual and reproductive development [8]. Based on these issues, the importance of assessing sexual maturation in studies involving adolescents is now widely accepted [9,10].

Therefore, the objective of the present paper was to determine body fat percentiles and cutoff values for excess overweight according to gender, age, and sexual maturation of a population in adolescents in the city of São Paulo, Brazil.

## Methods

The adolescents involved in this study took part in the “Nutritional Profile of Public and Private School Adolescents in São Paulo” study, a segmented population-based study that involved anthropometric data collection and questionnaires.

The original study assessed 8,020 adolescents in 43 randomly selected public and private schools from different regions of São Paulo in 2004 and 2005. Because the assessment of sexual maturation, which was vital to the present study, was not permitted at all of the schools, 4,690 adolescents (58.48% of the original study sample) aged 10–15 years were eligible for this study. The subjects were enrolled in grades 5–8 in 31 schools in São Paulo (25 public and 6 private). Girls accounted for 54.5% of the study sample.

The schools were randomly selected after considering the number (32 public and 11 private) and proportions (3:1) of public and private schools in each region. However, because we needed to exclude schools that did not include all of the variables necessary for the present study, the final ratio of public to private schools was about 4:1. Although there was greater opposition to assessing sexual maturation at private schools, we also encountered this problem at public schools in several regions of São Paulo. Therefore, the random selection process established in the initial study remains. The schools included in this study were those that received authorization from their administrations. Only adolescents whose parents or guardians provided written consent were allowed to participate in the study.

Adolescents who satisfied the following criteria were included in this study: (1) age 10–15 years; (2) complete data for weight (kg), height (m), and triceps and subscapular skinfold thicknesses data to assess body fat percentages; (3) assessment of sexual maturation; and (4) absence of clinical or functional limitations. Pregnant adolescents were excluded from the study.

This cross-sectional study was conducted in accordance with the ethical principles for research involving humans, complying with Resolution CNS 196/96. The study was approved by the research ethics committee of the Federal University of São Paulo (CEP no. 0977/03).

Anthropometric assessments were coordinated by a team of four researchers, three nutritionists, and a physical education teacher, all of whom were post-graduate students. They were trained in the techniques and standardization of the methods used, and had previously participated in a pilot study involving >2,000 adolescents.

Body mass measurements (kg) were obtained using a Seca portable digital scale with a capacity of 150 kg. The adolescents were weighed while standing on the platform without shoes and wearing light clothing in a steady position with their arms relaxed beside their bodies [11].

Height was measured using a Seca stadiometer at a 90° angle to the floor and according to the parameters established by Jelliffe [12] and the World Health Organization (WHO) [11]. Body mass index (BMI) (kg/m^2^) was calculated using these data. WHO-proposed criteria [13] were used to assess nutritional status.

Triceps and subscapular skinfold thicknesses were measured in triplicate on the left side of the body to the nearest 0.1 mm using a Sanny scientific caliper, using techniques that were standardized and recommended by Lohman et al. [14]. The mean of the triplicate values was used in the analysis. Body fat percentage was calculated using equations developed by Slaughter et al. [15].

Tanner’s pubertal staging method [16] was used to determine sexual maturation through self-assessment techniques for breast development (B1, B2, B3, B4, and B5) for girls and genitalia (G1, G2, G3, G4, and G5) for boys. We validated this approach using the method proposed by Matsudo and Matsudo [17], and classified adolescents who reported being in B1 and G1 as pre-pubescent, those in B2–B4 and G2–G4 as pubescent, and those in B5 and G5 as post-pubescent.

For the present study, the adolescents were classified according to their age as being in early adolescence (10–12 years old) and middle adolescence (13–15 years).

### Statistical analysis

Results are presented as the mean and standard deviation. The Kolmogorov–Smirnov test [18] was used to verify that the data were normally distributed. Dependent variables (weight, height, body mass, BMI, and body fat percentage) were compared between boys and girls using analysis of variance with two factors (gender and age; as biological and chronological factors, respectively), followed by Bonferroni’s multiple comparison test [18]. The association between BMI and body fat percentage was done using Pearson correlation test. The distribution of body fat percentage was assessed using specific percentiles (3th, 5th, 10th, 15th, 25th, 50th, 75th, 85th, 90th, 95th, and 97th). The LMS (curve, mean, and coefficient of variation) method [19] was used to set body fat percentage cutoff points according to gender, with the following values: <3th = low body fat; ≥3th to <85th = appropriate; ≥85th to <95th = moderately elevated body fat; ≥95th and < 97th = elevated body fat; and ≥97th = very elevated body fat [20]. For all analyses, we used a significance level of P < 0.05 [21]. All analyses were done using Statistical Package for the Social Sciences (SPSS) software version 18.0.

## Results

A total of 4,690 adolescents participated in the study (2,555 girls and 2,135 boys). The mean height, weight, BMI, and body fat percentage were calculated for each age group and gender. Although the absolute BMI of adolescents aged 13–15 years was greater than that of adolescents aged 10–15 years (Table 1), the prevalence of excessive weight (overweight and obesity as defined by WHO standards) was greater in early adolescence (10–12 years) than in middle adolescence (13–15 years). The rates excessive weight in adolescents and middle adolescence were 26.71 and 21.46%, respectively in males, and 21.74 and 17.44%, respectively, in females (data not shown). When we analyzed the differences between early and middle adolescence, we found that the boys showed a significant reduction in body fat percentage with advancing age while the opposite was true for girls (Table 1). Overall, 45.75 and 38.75% of boys and 24.95 and 33.84% of girls aged 10–12 or 13–15 years, respectively, were classified as having excess body fat (data not shown).

**Table 1:** Body mass, BMI, and BFP of adolescents aged 10–15 years according to age group

**Gender**	**Age (years)**	**Height (cm)**	**Body mass (kg)**	**BMI (kg/m**^**2**^**)**	**BFP**
**Boys**	10–12 (n = 1082)	149.6 ± 8.1	43.0 ± 10.5	19,0 ± 3.5	20.0 ± 8.6
	13–15 (n = 1053)	163.7^†^ ± 9.2	54.8^†^ ± 12.7	20.0^†^ ± 3.8	18.9^†^ ± 8.1
**Girls**	10–12 (n = 1311)	151.6* ± 6.3	45.1* ± 10.8	19.4 ± 3.7	20.9 ± 7.4
	13–15 (n = 1244)	159.0*^†^ ± 6.3	52,8*^†^ ± 10.1	20.8*^†^ ± 3.5	23.0*^†^ ± 6.8

*Significantly different (P < 0.05) between boys and girls in the same age group; ^†^significantly different (P < 0.05) between the two age groups in the same gender.

Abbreviation: *BMI* Body mass index, *BFP* Body fat percentage.

Table 2 shows that there were no significant differences in body fat percentage between boys and girls aged 10–11 years. However, among subjects aged 12–15 years, mean body fat percentage was significantly higher among girls than boys (P < 0.001).

**Table 2:** Comparison of body fat percentages between boys and girls aged 10–15 years

	**Boys**			**Girls**			**P**
**Age (years)**	**n**	**Mean (DP)**	**Range**	**n**	**Mean (DP)**	**Range**	
**10**	93	20.92 (8.2)	6.0–51.3	117	20.7 (7.8)	8.1–48.2	0.88
**11**	457	20.20 (8.9)	4.6–58.9	527	20.2 (7.6)	4.3–58.8	0.99
**12**	532	19.82 (8.5)	4.4–57.2	667	21.5 (7.1)	6.7–48.9	<0.001
**13**	483	19.36 (8.5)	4.9–65.6	598	22.5 (7.1)	4.4–56.9	<0.001
**14**	323	18.16 (7.6)	4.7–50.9	400	23.4 (6.7)	7.5–51.2	<0.001
**15**	103	16.79 (6.0)	6.2–38.8	100	23.2 (5.3)	4.3–36.4	<0.001

Abbreviation: *DP*, Standard deviation.

Table 3 shows that pubescent and post-pubescent boys had lower body fat percentages than girls in the same pubertal stages (P < 0.001). However, there was no significant difference in body fat percentage among the pubertal stages in boys. In girls, body fat percentage increased significantly with increasing pubertal stage. We found that 29.56% of pre-pubescent boys and 28.75% of pubescent boys had high body fat percentages, as compared with 12.07 and 19.29% of pre-pubescent and pubescent girls, respectively. However, in the post-pubertal stage, girls presented with a higher body fat percentage compared with boys (37.33% vs. 23.53%; data not shown).

**Table 3:** Body mass, height, BMI, BFP of adolescents according to sexual maturation

**Gender**	**Sexual maturation**	**Height (cm)**	**Body mass (kg)**	**BMI (kg/m**^**2**^**)**	**BFP**
**Boys**	Pre-pub. (n = 115)	149.4 ± 8.7	44.0 ± 12.2	19.4 ± 3.8	21.0 ± 9.3
	Pub. (n = 2003)	156.8^†^ ± 11.1	49.0^†^ ± 13.0	19.6 ± 3.7	19.4 ± 8.3
	Post-pub. (n = 17)	169.1^‡§^ ± 13.3	57.5 ± 142	19.8 ± 3.1	16.8 ± 8.4
**Girls**	Pre-pub. (n = 116)	146.2 ± 8.9	38.4* ± 10.3	17.7* ± 3.5	18.5 ± 7.5
	Pub. (n = 2364)	155.5*^†^ ± 7.7	49.1^†^ ± 10.9	20.1*^†^ ± 3.6	21.9*^†^ ± 7.1
	Post-pub (n = 75)	158.5*^‡^ ± 7.0	57.2^‡§^ ± 10.1	22.6*^‡§^ ± 3.3	26.0*^‡§^ ± 6.4

*Significantly different (P < 0.05) between boys and girls in the same pubertal stage; ^†^Significantly different (P < 0.05) between the pre-pubescent and pubescent stages; ^‡^Significantly different (P < 0.05) between the pre-pubescent and post-pubescent stages; ^§^Significantly different (P < 0.05) between the pubescent and post-pubescent stages.

Abbreviation: *BMI* Body mass index, *BFP* Body fat percentage, *Pub* Pubescent.

Regarding nutritional status, although there was no difference in absolute BMI among the pubertal stages in boys, 33.91% of pre-pubescent, 23.61% of pubescent, and 17.65% of post-pubescent boys had excessive weight. The respective values in girls were 12.93, 19.42, and 37.33% (data not shown). These results indicate that excess weight was more common in pre-pubescent boys and post-pubescent girls, validating the higher body fat percentage in pre-pubescent boys compared with that in girls of the same pubertal stage.

We next developed gender- and age group-specific body fat percentiles (Table 4), as well as gender- and sexual maturation-specific body fat percentiles (Table 5).

**Table 4:** Body fat percentiles according to gender and age group

**Gender**	**Age group (years)**	**3th**	**5th**	**10th**	**15th**	**25th**	**50th**	**75th**	**85th**	**90th**	**95th**	**97th**
**Boys**	10–12 (n = 1082)	8.6	9.4	10.4	11.6	13.4	18.2	26.1	29.9	31.6	35.1	39.0
	13–15 (n = 909)	8.4	9.5	10.5	11.4	13.1	16.4	22.3	27.6	29.7	33.5	37.7
**Girls**	10–12 (n = 1311)	9.5	10.4	12.1	13.4	15.5	19.6	25.1	28.1	31.1	35.4	37.3
	13–15 (n = 1098)	11.4	12.4	14.7	16.2	18.1	22.4	26.3	29.2	32.3	35.4	37.1

**Table 5:** Body fat percentiles according to gender and sexual maturation

**Gender**	**Sexual maturation**	**3th**	**5th**	**10th**	**15th**	**25th**	**50th**	**75th**	**85th**	**90th**	**95th**	**97th**
**Boys**	Pre-Pub. (n = 113)	8.6	9.7	10.3	11.3	13.4	19.5	27.7	30.4	32.2	36.1	43.3
	Pub. (n = 1864)	8.5	9.4	10.5	11.4	13.1	16.9	23.8	27.9	30.9	34.1	37.5
	Post-Pub. (n = 14)	10.6	10.4	10.6	11.4	11.7	12.8	16.8	21.8	23.6	*	*
**Girls**	Pre-Pub. (n = 115)	8.1	8.5	10.2	11.1	13.0	16.7	21.5	24.8	28.1	34.0	35.9
	Pub. (n = 2227)	10.4	11.5	13.4	14.9	16.9	21.2	25.7	28.9	31.4	35.3	37.2
	Post-Pub. (n = 67)	13.8	15.0	17.7	18.9	21.1	25.1	30.0	34.8	36.8	36.5	37.6

Abbreviation: *Pub* Pubescent.

In subjects aged 10–12 years, percentiles of ≤50th were lower in boys than in girls, while the opposite was true for percentiles ≥75th. Among subjects aged 13–15 years, almost all of the percentiles were higher in girls than in boys (Table 4).

Table 5 shows that boys had higher body fat percentages in the pre-pubescent stage alone and that the body fat percentages of girls increased during puberty. The body fat percentiles according to sexual maturation in post-pubescent girls were greater than those calculated according to age. However, among boys in the same pubertal stage, they were smaller in the 25th percentile and above.

Table 6 presents the cutoff values for body fat percentages according to gender and age, while Table 7 presents the cutoff values according to gender and sexual maturation.

**Table 6:** Cutoff points for body fat percentages of adolescents aged 10–15 years according to gender

**Gender**	**Age (years)**	**Low (<3th)**	**Normal (≥3th to <85th)**	**Moderately elevated (≥85th to <95th)**	**Elevated (≥95th to < 97th)**	**Very elevated (≥97th)**
**Boys**	10	6.97 ± 1.25	18.72 ± 5.16	31.56 ± 1.47	36.05 ± 1.10	51.05 ± 0.38
	11	7.25 ± 1.35	17.76 ± 5.40	32.50 ± 2.37	40.62 ± 1.76	46.82 ± 4.37
	12	7.08 ± 1.19	17.74 ± 5.87	32.12 ± 1.10	36.02 ± 0.96	42.29 ± 5.17
	13	7.20 ± 1.04	16.98 ± 4.97	31.02 ± 1.83	37.91 ± 0.92	46.43 ± 7.15
	14	7.78 ± 1.36	15.96 ± 4.44	29.31 ± 1.75	34.28 ± 1.45	42.52 ± 5.18
	15	7.56 ± 1.43	15.68 ± 4.08	28.52 ± 1.88	33.12 ± 0.92	41.13 ± 5.13
**Girls**	10	8.56 ± 0.41	18.60 ± 4.69	32.54 ± 2.71	37.00 ± 0.77	44.53 ± 3.27
	11	6.80 ± 1.41	18.18 ± 4.60	30.90 ± 2.06	36.56 ± 0.77	42.10 ± 5.85
	12	8.97 ± 1.06	19.60 ± 4.52	31.48 ± 2.07	36.57 ± 0.65	41.59 ± 2.60
	13	9.02 ± 1.68	20.72 ± 4.30	31.95 ± 1.84	36.81 ± 0.68	43.13 ± 6.21
	14	10.41 ± 1.42	21.71 ± 4.32	32.97 ± 1.64	36.33 ± 0.46	42.04 ± 4.46
	15	13.18 ± 0.25	21.93 ± 3.62	30.48 ± 1.86	33.97 ± 0.03	36.09 ± 0.41

**Table 7:** Cutoff points for body fat percentages of adolescents aged 10–15 years according to gender and sexual maturation

**Gender**	**Sexual maturity**	**Low (<3th)**	**Normal (≥3th to <85th)**	**Moderately elevated (≥85th to <95th)**	**Elevated (≥95th to <97th)**	**Very elevated (≥97th)**
**Boys**	Pre-Pub.	7.44 ± 1.86	18.84 ± 6.69	32.54 ± 1.55	41.04 ± 2.35	48.50 ± 3.67
	Pub.	7.32 ± 1.17	17.05 ± 5.00	31.09 ± 1.56	36.30 ± 1.00	44.33 ± 5.68
	Post-Pub.	____	13.57 ± 2.43	24.18 ± 1.05	___	___
**Girls**	Pre-Pub.	7.86 ± 0.59	16.48 ± 4.46	29.52 ± 3.17	35.01 ± 0.11	41.82 ± 5.68
	Pub.	8.61 ± 1.56	20.03 ± 4.47	31.55 ± 1.80	36.20 ± 0.54	41.86 ± 4.84
	Post-Pub.	11.98 ± 3.05	24.90 ± 4.97	36.83 ±0.62	___	38.73 ± 1.10

Abbreviation: *Pub* Pubescent.

Among boys, the mean body fat percentages of the 85th, 95th, and 97th percentiles were 31.03 vs. 29.10, 37.19 vs. 34.84, and 45.98 vs. 42.82, respectively, for those aged 10–12 vs. 13–15 years. Among girls, the mean vales of the 85th, 95th, and 97th percentiles were 31.07 vs. 31.26, 36.07 vs. 35.41, and 42.29 vs. 40.36, respectively, for those aged 10–12 vs. 13–15 years (Table 6).

Body fat percentage classified as low or very elevated were not found in post-pubescent boys, and body fat percentage classified as elevated was not found in boys or girls. Among boys, the body fat percentages of the 85th, 95th, and 97th percentiles were higher when classified according to sexual maturation than the mean values for the ages of 10–12 or 13–15 years. For girls, although there was no significant difference in the mean body fat percentages according to age for the 85th percentile, the differences were greater when classified according to sexual maturation. The mean values for the 95th and 97th percentiles classified according to age approached those according to sexual maturation.

## Discussion

We present data and percentile curves of body fat percentages, based on skinfold thicknesses, for Brazilian adolescents aged 10–15 years. Although the measurement of skinfold thickness is considered very useful in epidemiological studies because of its ease of measurement and low cost, Deurenberg et al. [22] reported an error rate of 3–5% when it is used for pre-pubescent adolescents.

Body fat percentages can be used in obesity prevention as a specific assessment tool because BMI represents a single gross value for body fat, and does not differentiate between gender and pubertal stage. Therefore, the use of BMI may result in misclassification of obesity [23].

In this study, we confirmed that, in early adolescence (10–12 years old), there were no significant differences in BMI or body fat percentages between boys and girls. However, in middle adolescence (13–15 years old), boys had lower body fat percentages than girls, even though boys were taller and had greater body mass.

The mean body fat percentages of girls aged 10–12 and 13–15 years were very similar to those reported by Papandreou et al. [24]. However, our values were much lower than those reported by Ogden et al. [25], who analyzed body fat percentages in children and adolescents in the United States using dual-energy X-ray absorptiometry. The mean body fat percentages in boys and girls aged 10–12 years differed by 8 and 11%, respectively, for subjects aged 10–12 years, and by 5.5% and 9.3%, respectively, for subjects aged 13–15 years between the two studies. Although we must consider that the differences in methods used in these studies contributed to the different values, skinfold thickness is widely measured in population studies and in public health clinics. Therefore, the data presented in this study are very important for the development of body fat percentage screening tools.

In middle adolescence, the body fat percentages were higher in girls than in the boys in the pre-pubescent and pubescent stages. Similar results were reported by other authors [20,24,26-28]. These differences reflect physiological development, as girls gain more body fat while boys gain more muscle mass during puberty [29] because of the drastic hormonal changes that induce important modifications in growth, bone mass, and body composition. These modifications are associated with certain biochemical parameters—true “markers”—which regulate bone ‘turnover’ and leptin levels, reflecting changes in bone growth and fat mass, respectively [30,31].

To date, no Brazilian study has determined body fat percentages at different stages of sexual maturation, even though such data are vital during adolescence because of the marked variation in pubertal events among individuals of the same gender and age. Moreover, BMI was shown to increase in each stage of sexual maturation [32]. Thus, this study presents an important contribution to body composition assessment in adolescents, increasing the reliability of this parameter during adolescence.

When body fat percentages were distributed in percentiles, we found that our values were slightly higher than those reported by McCarthy et al. [20] and Kurtoglu et al. [26], but were lower than those reported Papandreou at al [24], who measured body fat percentage using bioelectrical impedance analysis. In the study by Ogden et al. [25], the body fat percentages were much higher than those reported in the studies cited above [20,24,26] and the present study, because the 95th percentile for body fat was >40% in boys and girls.

When we evaluated body fat percentage according to sexual maturation, we found that pre-pubescent boys had 11.4% more body fat than did girls. However, in the post-pubescent stage, the body fat percentage was nearly two times higher in girls than in boys (25.2% vs. 13%). According to Kurtoglu et al. [26], body fat percentage increases during puberty in boys and girls, but decreases in boys after puberty and remains constant thereafter. Although we only enrolled adolescents aged 10–15 years and the number of adolescents considered biologically mature was small, we did not observe this behavior in this cohort.

One limitation of this study is that, by separating the adolescents aged 10–15 years according to sexual maturation stage, most were classified as pubescent and that there were few pre-pubescent adolescents (predominately male) and post-pubescent adolescents (predominately female). The sample size could also influence our results. In future studies, it will be necessary to include a larger sample of subjects, and include subjects in all stages of sexual maturation.

Surprisingly, the aforementioned studies did not include assessments of sexual maturation. This is an important factor because we found minimal differences in the mean body fat percentages of the 95th and 97th percentiles among the pubertal stages in girls. These results suggest that excess body fat in the early stages of sexual maturation may persist, increasing the risk of obesity.

Although no consensus has been reached for the diagnosis of obesity based on body fat percentages, some authors have defined the 85th and 95th percentiles as excess body fat and obesity levels, respectively [20,26,33-38]. The mean values for these percentiles for adolescents aged 10–15 years were higher in our study than those reported by McCarthy et al. [20], Kurtoglu et al. [26], and Papandreou et al. [24], but were lower than those reported by Odgen et al. [25]. When we calculated body fat percentages according to sexual maturation, we found that the 85th and 95th percentiles were about 0.5% higher in pre-pubescent and pubescent boys than the values for boys aged 10–12 and 13–15 years. However, these percentiles were lower in pre-pubescent girls than in girls aged 10–12 years, but were higher in pubescent girls than in girls aged 13–15 years. These data demonstrate the impact of sexual maturation on body composition, and the importance of assessing sexual maturation in adolescents. Because the boys in our population were more frequently classified as overweight, consistent with our clinical experience, we consider it important to use these data when assessing body composition of adolescents.

## Conclusion

To our knowledge, this is the first study to determine body fat percentages of adolescents according to gender and sexual maturation, and demonstrate the important of assessing pubertal stage because the age at which each stage is reached can vary considerably. Because sexual maturation has a significant influence on body composition, cutoff values that only consider chronological age are inadequate for assessing obesity in adolescents. Thus, this study presents a significant contribution showing the influence of sexual maturation on body fat development, and that assessing sexual maturation is essential to better understand the characteristics of adolescent obesity. Other studies using homogenous samples of all pubertal stages are necessary to confirm our findings. Nevertheless, the values and percentile curves for the body fat percentage of adolescents according to age, gender, and sexual maturation will help doctors and other health advisors to identify and prevent obesity in adolescents, and lower the risk of obesity and its associated health problems in later life.

## Abbreviations

BFP: Body fat percentage; WHO: World Health Organization; BMI: Body mass index; LMS: (L) curve. (M) mean and (S) coefficient of variation.

## Competing interest

The authors have no conflicts of interest to declare.

## Authors’ contributions

IPC conceived, designed, and implemented the study, collected and interpreted data, and helped to write and revise the manuscript; GLMF performed statistical analyses, interpreted the data, and helped to write and revise the manuscript; ACSVS helped to implement the study and to write the manuscript; MAZP was responsible for the data collection, helped implement the study, and helped to write the manuscript; MF was responsible for coordinating original study and contributed to the intellectual content; MSSV interpreted the data, and helped to write and revise the manuscript. All authors have read and approved the manuscript for publication.

## Authors’ information

All authors are registered on the Lattes platform.

## Pre-publication history

The pre-publication history for this paper can be accessed here:

http://www.biomedcentral.com/1471-2431/13/96/prepub
